# Going home? An ethnographic study of assessment of capacity and best interests in people with dementia being discharged from hospital

**DOI:** 10.1186/1471-2318-14-56

**Published:** 2014-04-23

**Authors:** Marie Poole, John Bond, Charlotte Emmett, Helen Greener, Stephen J Louw, Louise Robinson, Julian C Hughes

**Affiliations:** 1 Institute of Health and Society, Newcastle University, The Baddiley-Clark Building, Richardson Road, Newcastle upon Tyne NE2 4AX, UK; 2 Centre for Mental Health Law, Northumbria Law School, Northumbria University, City Campus East, Newcastle upon Tyne NE1 8ST, UK; 3 Gateshead Health NHS Foundation Trust, Bensham Hospital, Saltwell Road, Gateshead NE8 4YL, UK; 4 Newcastle upon Tyne Hospitals NHS Foundation Trust, Freeman Hospital, Newcastle upon Tyne NE7 7DN, UK; 5 Northumbria Healthcare NHS Trust, North Tyneside General Hospital, Rake Lane, North Shields, Tyne and Wear NE29 8NH, UK; 6 Institute for Ageing and Health, Newcastle University, Campus for Ageing and Vitality, Newcastle upon Tyne NE4 5PL, UK

**Keywords:** Capacity, Best interests, Decision-making, Dementia, Ethnography, Hospital discharge, Mental Capacity Act, Residence capacity

## Abstract

**Background:**

A significant proportion of patients in an acute hospital is made up of older people, many of whom have cognitive impairment or dementia. Rightly or wrongly, if a degree of confusion is apparent, it is often questioned whether the person is able to return to the previous place of residence. We wished to understand how, on medical wards, judgements about capacity and best interests with respect to going home are made for people with dementia and how decision-making around hospital discharge for people with dementia and their families might be improved. Our research reflects the jurisdiction in which we work, but the importance of residence capacity rests on its implications for basic human rights.

**Methods:**

The research employed a ward-based ethnography. Observational data were captured through detailed fieldnotes, in-depth interviews, medical-record review and focus groups. Themes and key issues were identified using constant comparative analysis of 29 cases. Theoretical sampling of key stakeholders was undertaken, including patients with dementia (with and without residence capacity), their relatives and a range of practitioners. The research was carried out in three hospital wards (acute and rehabilitation) in two hospitals within two National Health Service (NHS) healthcare trusts in the North of England over a period of nine months between 2008 and 2009.

**Results:**

Our analysis highlights the complexity of judgements about capacity and best interests in relation to decisions about place of residence for people with dementia facing discharge from hospital. Five key themes emerged from data: the complexity of borderline decisions; the requirement for better understanding of assessment approaches in relation to residence capacity; the need for better documentation; the importance of narrative; and the crucial relevance of time and timing in making these decisions.

**Conclusions:**

We need: more support and training for practitioners, as well as support for patients and families; clarity about the information to be imparted to the person with dementia; more advocacy for people with dementia; appropriate assessments embedded in routine clinical practice; the patient with dementia to be centre-stage; and properly resourced step-down or rehabilitation units to facilitate timely and good decision-making about place of residence.

## Background

For many people with dementia admitted to hospital a question is raised, rightly or wrongly, about whether they will be able to manage at home. These concerns may not always be shared by patients, who may desire to return home in spite of known or perceived risks. Clinical teams may feel this calls into question the person’s ability to make their own choices about living arrangements on discharge. In such circumstances the person’s decision-making capacity (i.e. specifically residence capacity^a^) should be assessed and, if the person lacks capacity (sometimes called competence), a decision will need to be made on his or her behalf. In England and Wales, decisions about capacity are governed by the *Mental Capacity Act 2005* (MCA) and if capacity is lacking a decision must be made in the person’s ‘best interests’.^b^ But beyond the jurisdiction of the MCA such judgements will have to be made too, in any country that takes the liberty of its citizens seriously. This paper reflects the perspective of those working with the MCA, but its findings and implications will be relevant worldwide.

These decisions and the evaluative issues they raise can be conceptually complex [1]. But such decisions are a daily occurrence in hospital wards. As the prevalence of dementia increases in the UK and worldwide [2], the number of patients in hospitals with dementia is also likely to increase. In the UK, the need to improve hospital care of people with dementia, where they occupy a quarter of hospital beds, is well recognized [3]. A recent study in London showed that 42% of those over 70 years admitted to hospital had dementia [4]. But more people with dementia in hospital means potentially more people being discharged to long-term care. A report by the Alzheimer’s Society in the UK suggested that ‘Over a third of people with dementia who go into hospital from living in their own homes are discharged to a care home setting’; 60% of people with dementia in the study were admitted to hospital from their own homes, but only 36% returned there [5].

Discharge from hospital, therefore, is a critical process for older people with dementia. Yet people with dementia and their carers are not involved in decision-making as much as they would like to be [5]. Whilst professionals profess to be familiar with the MCA, they find it difficult to put its principles into practice [6]. They are particularly concerned about risks. The importance of a capacity assessment, as part of a permissive attitude towards risk, has been emphasized in the UK: ‘… an activity or an arrangement should be permitted or respected unless risk analysis, including an assessment of capacity if this is in doubt and determination of best interests, shows it should not’ [7].

The aim of our research was to understand how residence capacity and consequent best interests for people with dementia are decided in acute and rehabilitation hospital settings. In this paper the focus is largely on the determination of capacity, but the issue of best interests is implicit in that the capacity determination often seemed to predict the outcome, which was deemed to be in the person’s best interests. The research provides a direct comment on how the MCA works in practice, which is currently a topic of investigation in the UK by the House of Lords [8]; but the broader principles will be relevant worldwide where the rights of people with dementia are of increasing concern as populations age.

## Methods

**Figure 1:**
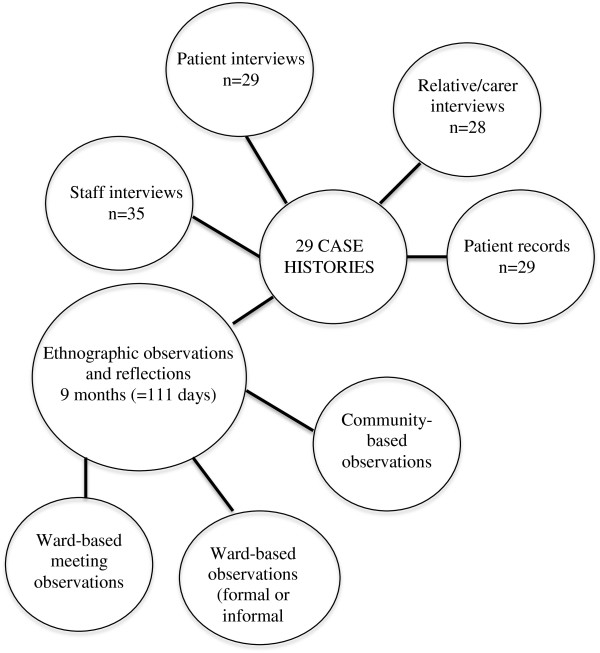
Overview of ethnography.

We used an ethnographic approach [9] supported by social constructionist theory [10]. Thus, we observed interactions from different perspectives and in different settings. The different sources of data collected during the fieldwork on the wards are depicted in Figure 1. The fieldwork was summarized by the construction of detailed case histories. The case histories formed the basis of analysis, which was also informed by a literature review [1]. Finally, focus groups provided reflection on the emerging findings of the project. In keeping with standard practice in qualitative research, the analysis of data started immediately, was on-going and iterative [11].

The social constructionist approach suggests the need for reflexivity. We were aware of our own personal and professional backgrounds, which would tend to colour our judgements about observations and the emerging data. The stance of the whole research team was clearly on the side of enhancing and protecting the rights and dignity of people with dementia. Nevertheless, there was enough clinical and research experience amongst the team to make us acutely aware of the pressures on both professionals and family carers in the situations that arose in the context of difficult decisions about discharge on busy medical wards. The main researcher undertaking the ethnographic observation (MP) was naïve concerning the environments in which she was working, although there was a period of familiarization before formal observation began. She (MP) maintained a strictly objective stance as a researcher during ward meetings, for instance, if her opinion was sought. Some of the clinicians in the research team have worked, and do work, as colleagues with the professionals on the wards where observations were carried out. Our opinion of the clinical skills and personal qualities of these professionals was, and is, of a high order. Creative tensions between the professional backgrounds of the research team – reflecting the different approaches of social science, the law, medicine and psychiatry – were sometimes in evidence as a constructive part of the analysis of data, for instance during data workshops.

Ethical approval was granted by the NHS regional ethics committee (Newcastle and North Tyneside 2 Research Ethics Committee Ref No:08/H0907/50). The details of the ethics procedures adopted during the study were as follows.

Where possible and relevant valid written consent was sought. Everyone working or receiving care on the wards observed was given an information leaflet (targeted to their situation, i.e. whether they were professionals or patients) about the project. Posters about the research were displayed in the wards. Where the clinical team felt it might be appropriate to involve a patient in the research, the person was approached by the clinical team initially, given an information sheet and asked if the researcher could discuss participation with them later, after time for reflection. If it was felt that the person lacked capacity to consent to participate, a personal or nominated consultee was sought in accordance with the requirements of the MCA (Section 32) and guidance from the Department of Health [12]. Even so, patients who lacked capacity to participate in the research were still given a simplified information sheet and were approached to seek some form of assent and permission for the researcher to approach a relative of their choice. At the time of the first interview both patient participants and their family carers were asked to consent to a follow-up interview at three months after the date of discharge from hospital. No observations were recorded without valid consent. Valid written consent was also obtained from those who participated in focus groups. All those in the research team involved in patient contact or with access to their data had either permanent or honorary contracts with the NHS Trusts involved, whose Research and Development departments had given permission for the study; Caldicott Guardian approval (i.e. the process in the NHS which governs the use of confidential information) was also granted.

Theoretical sampling of patients and staff took place to ensure a broad spectrum of situations and relationships [13]. Inclusion criteria were that the patients should have a presumed diagnosis of dementia or cognitive impairment, whether or not made formally, and that a question about place of residence should have been raised. We excluded people with a diagnosis of delirium. Once data saturation was achieved, 29 patient participants were included, based on a number of key characteristics: stage of dementia and cognitive impairment; the presence of informal carers and support; formal social support; co-morbidities; and pre-admission living arrangements.

Fieldwork was conducted in three general hospital wards (orthogeriatrics, care of the elderly and rehabilitation) in two hospital trusts in the North East of England. Data were collected over a nine-month period, between June 2008 and June 2009, which included a three-month analysis period. Data comprised written field notes or tape recordings, which were transcribed and anonymised.

Observations were predominantly ward-based and recorded as near to the moment of observation as possible [14]. This allowed us to investigate how the MCA was being applied in routine clinical practice. General everyday activities as well as more specific activities were observed, such as multidisciplinary team (MDT) meetings, ward rounds, planning meetings/case conferences and ad-hoc meetings between carers and doctors. A small number of observations were conducted in the community, as well as at follow-up three months post-discharge.

A review of medical records for each patient supplemented observational data. Records from all disciplines – including medical, nursing, therapy, social work and old age psychiatry – provided insight into events, which were often unobserved, and added clarity concerning the timing of events in the course of the person’s admission.

In-depth interviews elicited personal perspectives and experiences around the process of decision-making and the effects and consequences of the judgements made. Informal face-to-face and telephone discussions with patients, carers, and professionals took place. There were also interviews with professionals in relation to their understanding of the MCA. Interviews were conducted around the time of discharge; and, if possible, three months post-discharge with patients and carers.

Reflections on observations and interviews were recorded. Discussion of the content of these notes informed how the research continued and generated ideas for exploration as part of the overall analysis.

To draw on a broader base of perspectives and experiences, separate focus groups were held with carers of people with dementia and with health and social care professionals. Using hypothetical cases derived from ward-based observations, the groups discussed how discharge decisions could be improved.

Data workshops (involving MP, JB and JCH) allowed us to discuss the themes emerging from the coding of the transcribed data and to construct a coding framework for the further analysis of all data. Concepts were developed by writing memos (summarizing cases) to explore them in more detail in context [15]. The data were managed using NVIVO software [16].

Constructing case histories for each case enabled the synthesis of multiple and varied data (see Figure 1) into a coherent patient journey from pre-admission to three months post-discharge. Each case incorporated a range of perspectives and described in some detail the experiences of patients and their relatives and how assessments of residence capacity and consequent judgements about best interests were made. Comparing and contrasting the cases using constant comparative methods [17] allowed us to identify commonalities and differences across cases and to highlight key areas for potential improvement.

## Results

A total of 92 interviews with key stakeholders were conducted alongside observations (see Figure 1). The professionals represented a broad range of disciplines and experiences including staff from junior to senior levels: doctors, occupational therapists (OTs) and physiotherapists, nurses, social workers, an Independent Mental Capacity Advocate (IMCA), plus specialist services, e.g. old age psychiatry. The research team (JB, JCH, MP, HG) facilitated four focus groups: three for professionals (n = 22) and one made up of family carers (n = 3) and voluntary agency carers’ supporters (n = 2). This expanded the professional perspectives to include general practice (including a trainee), chaplaincy and nursing homes.

The background demographic details are provided in Table 1, which also shows how many people were judged to have capacity by the clinical team, how many formal assessments of capacity were carried out and to where participants in the study were discharged. It also records that only 20 out of the 29 had a formal diagnosis of dementia, although they all had cognitive impairment.

**Table 1:** Demographic and background details of 29 participants, including capacity assessments and place of discharge

Age	Mean = 83 (range 69–92)
Gender	Female = 16; Male = 13
Ethnicity	White British = 28; White European = 1
Location	Acute =20; Rehab =9
Average length of stay	Acute ward = 35 days (range 13–59 days)
	Rehabilitation ward = 87 (range 29–157 days)
Mini-mental state examination (MMSE) scores (see Table 2 for details)	Range 6–30
Diagnosis of dementia	n = 20
Formally recorded capacity assessments regarding place of residence^1^	n = 14
Clinical judgements of capacity regarding place of residence	Capacity = 13; Lacks capacity =16;
Discharge outcome	Home = 10; Care = 18 (Nursing = 9, Res = 9); Deceased = 1

^1^Table 1 notes the formally recorded assessments of capacity. This simply means that the results of assessments of residence capacity were written in the medical, nursing or social work notes. In this sense an informal assessment would be one where staff had formed the opinion that someone had or did not have capacity but this was not recorded in a legal document, i.e. not recorded in health or social care records.

Table 2 gives details of the 29 patients selected for interview whose cases then formed the basis of our analysis. This includes the average Mini-Mental State Examination (MMSE) [18] scores, made up of various scores by various professionals taken over the course of the person’s admission. In 20 cases, the professionals judged that the person’s residence capacity was uncertain. We have referred to these cases as ‘borderline’. The cases were defined in this way by the clinical teams at the time. But the notion of ‘marginal’ capacity (or competence), which we take to be equivalent to our notion of ‘borderline’ capacity, has been around for some time [19]. These borderline cases were equally distributed between those who were finally deemed to have capacity and those who were deemed to lack it. The mean of the average MMSE scores of those judged to have capacity was 20 (range: 14–28; n = 12); the mean of the average MMSE scores of those judged to lack capacity was 15 (range: 3–30; n = 15). In two cases there was no MMSE performed during the admission. At discharge, 10 returned to their homes, of whom only one was felt to lack capacity. Eighteen were discharged to care homes (one died in hospital), of whom only four were judged to have residence capacity; and in two capacity was regarded by professionals as doubtful. Hence, almost all those who returned to their homes were judged to have capacity and almost all those who lacked capacity went into care homes.

**Table 2:** Characteristics of 29 patients with destinations on discharge in alphabetical order, but split into those with capacity (in top portion) and those without (in bottom portion); names are fictitious and randomly chosen according to pre-determined schema

**Patient**	**Age**	**Living arrangements prior to admission**	**Average MMSE score (range)**^**1**^	**Capacity decision (‘B’ implies thought to be borderline)**	**Discharge destination**
Mrs. Bailey	90	Alone, home	18 (15–20)	Capacity (B)	Nursing Care
Mr. Cook	91	Alone, home	20 (20)	Capacity (B)	Home
Mrs. Friar	79	With husband, home	15 (15)	Capacity (B)	Home
Mrs. Gardiner	79	Alone, home	24 (20–26)	Capacity (B)	Home
Mrs. MacVicar	76	Alone, home	22 (19–24)	Capacity (B)	Nursing Care
Mrs. Mason	92	Alone, home	23 (20–28)	Capacity	Home
Mr. Mills	80	Alone, home	21 (14–26)	Capacity (B)	Home
Mr. Miner	74	With wife, home	Not assessed	Capacity (B)	Home
Mr. Priestly	84	With wife, home	18 (18)	Capacity (B)	Home
Mrs. Porter	69	Alone, Sheltered Accommodation	19 (17–20)	Capacity (B)	Residential Care
Mrs. Shearer	88	Alone, Sheltered Accommodation	21 (18–24)	Capacity	Home
Mr. Saddler	92	With son, home	14 (14)	Capacity (B)	Nursing Care
Mr. Walker	79	Alone, Sheltered Accommodation	21 (16–25)	Capacity	Home
Mrs. Baker	89	Alone, home	12 (11–15)	Lacked capacity (B)	Home
Mrs. Butler	74	Alone, home	9 (5–14)	Lacked capacity	Residential Care
Mrs. Carter	90	Alone, Sheltered Accommodation	9 (9)	Lacked capacity (B)	Residential Care
Mr. Coleman	82	With wife, home	19 (17–21)	Lacked capacity (B)	Nursing Care
Mr. Collier	74	Alone, home	28 (26–30)	Lacked capacity (B)	Residential Care
Mr. Day	91	Alone, home	14 (14)	Lacked capacity	Deceased
Mr. Fisher	82	With wife, home	Not assessed (8 prior to admission)	Lacked capacity	Residential Care
Mrs. Miller	90	Alone, Sheltered Accommodation	13 (11–14)	Lacked capacity	Nursing Care
Mrs. Parker	78	Alone, home	13 (13)	Lacked capacity (B)	Nursing Care
Mr. Ryder	87	Alone, home	12 (10–13)	Lacked capacity	Nursing Care
Mrs. Salter	88	Alone, home	7 (7)	Lacked capacity (B)	Residential Care
Mr. Shepherd	89	Alone, home	20 (20)	Lacked capacity	Nursing Care
Mrs. Tanner	85	Alone, Sheltered Accommodation	13 (8–18)	Lacked capacity (B)	Nursing Care
Mr. Tyler	83	Alone, home	15 (15)	Lacked capacity (B)	Residential Care
Mrs. Woodward-Jones	80	Alone, home	22 (18–24)	Lacked capacity (B)	Residential Care
Mrs. Wright	91	Alone, home	19 (19)	Lacked capacity (B)	Residential Care

^1^Table 2 records MMSE scores as averages, with the range also supplied. But these scores must be regarded with caution. The MMSE was used at different times during the person’s admission by different staff from various professional backgrounds. We are aware of at least one case in which the MMSE may have been used inaccurately; but there may have been other cases. Of course, a single score on a MMSE taken early in the person’s admission, when there may have been persisting confusion relating to the cause of the admission, cannot be compared to several MMSE scores taken late in an admission.

Most of the carers were daughters (n = 13) or sons (n = 10). There were four spouses (three wives and one husband). Other main carers were daughters-in-law (n = 3), a nephew and three friends. Some were interviewed twice, both at the time of discharge from hospital and three months later.

The analysis of ethnographic data within a social constructionist perspective involves both description of primary data and the interpretation of these data by the research team. Consequently, compared with quantitative paradigms, the presentation of results is more discursive. Our observational and interview data show how the relevant judgements about residence capacity are complex for all concerned, and can be considered in connection with five themes: borderline capacity; assessment approaches; documentation; narrative; time and timing.

### Borderline capacity

Healthcare professionals conceptualised the capacity of patients to make their own discharge decisions as either clear-cut or complex. Clear-cut cases seemed to the teams straightforward with a good deal of consensus around the requisite capacity. One consultant talked in an interview of it sometimes being ‘blindingly obvious’ that a person has or does not have capacity – which can be judged by the whole team – whereas, when this was not the case, the consultant felt it necessary personally to undertake the assessment. Borderline cases, however, which were not uncommon, were more complex. One consultant, at interview, suggested that about a third of patients fall into each group: clear-cut capacity, borderline capacity and clear-cut incapacity. In borderline cases, capacity was often considered to be marginal or fluctuating. As well as multiple assessments, they often entailed second opinions from, for instance, old age psychiatry. Planning meetings were more complex and case conferences were usually held to seek the views of relatives. Hence, borderline capacity led to resource-intensive discharge planning, which included an increase in the length of in-patient stays.

In the extract below it is clear that the occupational therapist (OT) changed her view about Mrs. MacVicar’s (see Table 2) residence capacity and finally decided that she lacked it; a formal assessment, however, concluded that she had the requisite capacity.

INT … and do you think that Mrs. MacVicar had the capacity in your opinion to make that decision about her discharge?

OT I think to start with. She probably did to start with, however at the end when she ended up going into nursing care she didn’t have capacity. There’s no way she did, because one minute you would speak to her about it and she would say, “Oh yeah I think they’re sending me somewhere, I’m going to stay with my husband”, and then she would say, “Oh but my husband is still at home, but I haven’t seen him recently”. And so she just was like she kept to-ing and fro-ing with what was going to happen with that. Even on the day she was going, because she said like the week before “Oh yeah I’m quite happy about that and I think that’ll be good because I don’t think I’ll manage at home”, and then when she went like a couple of days before she thought she was going home and then when she was going she was saying, “Oh I’m not too happy about this”, so kept kind of changing her mind on things and stuff. So she just didn’t have, she didn’t know where she was a lot of the time towards the end of being in here, so she definitely didn’t have capacity in the end I don’t think.

Interview with OT 02sC-1305

The complex nature of borderline capacity seems to stem from the role that values play in these judgements compared with the more straightforward clear-cut cases in which capacity can be treated more factually. When these judgements are factual, they more readily square with the requirements of the MCA. But many cases involve value judgements and values are more prominent when they are diverse and conflicting [1,20]. For example, in the case of Mr. Collier the issue was that his home was squalid. But judgements about how squalid a place has to be before someone is not allowed to return home are obviously evaluative. Nevertheless, such judgements appear to have an impact on decisions about both capacity and best interests. Of course, it is also true that, even if a case were not borderline, even if the judgement about capacity or incapacity were to be straightforward, this does not mean that the discharge from hospital would be straightforward. In the case of Mr. Ryder, for instance, the discharge itself was complicated because of intra-familial tensions; but this is simply another manifestation of the importance of diverse values and values complexity in clinical practice.

### Functional versus outcomes approaches

By contrasting interviews with observations, we uncovered some ‘mismatch’ in terms of theory and practice with regard to healthcare professionals’ appreciation of the difference between a *functional* and *outcomes* approach to the assessment of capacity [21]. There was little doubt that potential outcomes, as predicted by others (professionals or family), influenced capacity judgements. The perception of risk, in particular, affected assessments of the person’s capacity. If the person did not agree with the MDT, he or she was likely to be deemed to lack capacity.

Recognizing that agreement with the MDT tends to mean that capacity is not assessed and that worries about outcome tend to drive assessment, the liaison psychiatry nurse below opined that capacity assessments might be better if carried out routinely rather than only when a difficult decision is required.

INT: *I think at the moment we’ve kind of got stuck with thinking, ‘Right, what do we ultimately want for this person…’. There’s the patient, that’s what we want for the person and how do we get there, rather than going through a nice routine process. Um ...... I suppose every patient who came on this ward, for instance, if their capacity just now was assessed, regardless of what the outcome’s going to be, it would show that we’re actually doing it routinely, rather than just when we need to do it, because we want to make a decision that the person’s not going to like.*

**Interview with Psychiatry Liaison Nurse** ***01BsG-1510***

This theme also emerged in a focus group with professionals. In the extract below a nurse assessor (who determines the level of care a person will need after discharge from hospital) was considering a hypothetical case, in which a mother (Mrs Black) wished to go home but her daughter was concerned about the level of risk. The nurse assessor is aware of the importance of understanding the person’s functional processing, but worries about risk seem to predominate.

NURSE: I think my views were kind of around the acceptance of the risks, the risks of going home, against the daughter’s viewpoint on the risk of going home; why she was concerned about it and how Mrs Black actually processed that; so not processing the decision about the capacity but processing the concerns about the risk, how she would rationalise those.

FACILITATOR: Yeah, why do you think that’s so important?

NURSE: I just think it’s kind of an indicator about how or what her processes are. You know we meet obviously a lot of people who are about to be discharged from hospital and quite often their ultimate aim is to go home, kind of forsaking all other outcomes, it’s to go home. It’s the most desirable outcome for them … so I guess it’s about trying to get into the mindset about how they’re processing that. Is it just kind of this nirvana of going home or is it against everything else or is it about: “Well I know what the risks are and I’m prepared to accept that with assistance or without assistance”.

**Professional Focus Group** ***280409:259–277***

Even when pressed on exact criteria involved in an assessment of capacity under the MCA, the same nurse was keen to emphasize the importance of a broader view.

FACILITATOR: … but should the capacity assessment just be the ticking of those four boxes about recall, understand, weigh up and communicate?

NURSE: Personally I think any assessment has to be a holistic assessment drawing in as much information as you possibly can from the sources that are available …

FACILITATOR: Well I’m tempted just to say why, why must it be a holistic?

NURSE: I think because you’re looking at a person at the end of the day. You know, you’ve got to look at everything that’s going on for them and with them and to them and what other people’s viewpoints are about that, to put it all together. I guess that’s going to make the job of the person that’s doing the capacity assessment very difficult and very long, but that doesn’t necessarily make it a bad thing. And that argues for this fuller picture approach and those four words … It depends what you mean by “recall”. By that do we mean, can you tell the story in such a way that you understand the risks associated with going home? It’s a much bigger question than, you know, a little ticky-box recall things: it’s the fuller picture, understanding of the fuller picture isn’t it?

**Professional Focus Group *280409:759–779***

The nurse went on to give a very clear description of the sort of clinical concern that can arise for professionals when the legal criteria for capacity are strictly applied. In doing so the nurse demonstrated that outcome, epitomised in terms of risk, is prone to higher consideration than the functional assessment of capacity in the minds of some practitioners.

NURSE: I know for a fact [of] somebody that has been assessed as having capacity because they passed the capacity test but she is being cared for very precariously should we say and not, well, it’s not in the least bit safe … It’s my feeling that the person that applied the capacity test had a look at the kind of process and evaluation, communicating, retaining the information thing and said, “OK, that’s absolutely fine”. They looked at this person once over a very short period of time … I wouldn’t challenge that she did have capacity at that time and that’s what the Mental Capacity Act is all about. But when you look at the longer term and you look at the risks that are involved and kind of the holistic picture, the whole picture of what’s actually involved, it was probably still a very, very unsafe and very unwise decision.

**Professional Focus Group** ***280409:783–797***

The extent to which the ‘factual’, more functional, approach was uncomfortable for practitioners was clearly enunciated by a chaplain who attended a focus group.

CHAPLAIN: I think I’d talk about people’s familiarity with personality and idiomatic phrasing, a sense of humour, and those kind of qualities which if you’re just taking a very factual approach to them and a very kind of neutral approach to assessment you might well miss. And those are the kind of things that are very difficult to decide just on a one-off encounter, a one-off assessment based approach; but I think that the kind of things that come over a period of time of being with somebody and listening attentively to them, so that they feel that they’re being heard, so the language they use, the nuances and all the things that lie behind … I think we really only get to know each other… through relationships and the way in which we express ourselves.

**Professional Focus Group *280409:542–551***

Similarly, in our discussions in a focus group with family carers of people with dementia, when the issue of capacity was specifically addressed, the natural inclination of the carers was to stress safety. One carer defined capacity as being capable of making a decision that was safe. The daughter of a mother with dementia talked of taking into account everything that a *reasonable* person would take into account; and another family carer stated that she could not take risks (Carers’ Focus Group *290409:12.45-12.53*).

Again to use the case of Mr. Collier, a social worker discussed some of the problems of assessing his capacity. It was apparent that his lack of engagement with the perception of risk – as judged by the professionals – was enough to call into question his capacity. Mr. Collier was resistive to the recommendations of the team, which was interpreted as a lack of insight sufficient to affect his capacity. Hence, what was really driving the capacity assessment were worries about outcome rather than mental functioning.

He’s got quite a good façade when you talk to him but I think if you get underneath that he really doesn’t have the capacity to understand what is safe and what isn’t safe. We’ve offered him carers at home and he refuses them. He confabulates, he just “Well, we’ll do it next week or the week after”. We’ve offered him the opportunity to consider re-housing, sheltered accommodation, and it’s the same response basically: “Not just yet but at some point we will”. So engaging him at any meaningful level has been quite difficult.

Interview 02sJ-0206: Social Worker

The importance of the distinction between a functional and outcome assessment of capacity is demonstrated by Mr. Collier’s having been judged to lack capacity: he was discharged to residential care, despite his express wish to go home. At follow-up, he said that he remained unhappy about the decision and felt that he had been ‘tricked’, which demonstrates the importance of these assessments in connection with deprivation of liberty [22].

### Documentation

Given the importance and complexity of many assessments of residence capacity and best interests, proper documentation is essential: clinically, ethically and legally. A review of medical and other ward-based records revealed significant differences in terms of content and quality with respect to both the determination of residence capacity and judgements about best interests. The final capacity decision was only recorded in the notes of two-thirds of the patients. Entries ranged from single sentences through to detailed and descriptive accounts. On one ward, a specific proforma relating to the key criteria of the MCA was being piloted.

In all cases, the narrative of the decision-making trail was dispersed throughout the medical and other records, such as social work notes, which were not always kept on the ward. Routine capacity decisions were recorded in notes taken at MDT meetings or during ward rounds. Different approaches were taken by different disciplines. For example, entries by old age psychiatry and social work teams were more likely to be detailed and encompass core principles of the MCA in comparison with the entries of physicians.

Below are examples (with minor modifications to preserve anonymity) of how residence capacity assessments were recorded in three different ways for Mrs. Gardiner.

Example 1: Entry in clinical notes

<Date>: Ward round – Dr (name), 12 pm, Assessment of capacity. Present: Dr (name), Ward Manager G, Dr A (SpR). Capacity form completed, does have capacity; having recurrent [urinary tract infections]; for prophylactic antibiotics and plan is a bladder scan.

Example 2: Entries on capacity proforma (stored separately to above clinical note, where text in italics represents entry on form by consultant)

<Date>

Assessor: [*Dr. (name) consultant physician*].

Impairment of brain or mind due to: *? dementia*

Decision to be made: *To accept residential care.*

Use the patient’s own words wherever possible to support outcomes. Is patient able:

1) To understand and to restate each element of information in his or her own words? *Yes. She admits that her memory is not so good now afraid to go back home and live on her own*

2) To retain this information? *Yes*

3) To use and weigh this information? *Yes*

4) To communicate the decision reached? *Yes, … was able to communicate that she would like to continue alternative placement.*

Example 3. Entry in clinical notes by old age psychiatry liaison nurse

<Date>: Old Age Psychiatry liaison, asked to see re level of care. [Mini-Mental State Examination] 23 out of 30, 6 out of 10 for orientation, 5 out of 5 for concentration, 1 out of 3 for recall. Depression screen is negative. Patient was unable to give a reasonable history of her circumstances. On occasions was disorientated and confused, i.e. said she had just been to the shop for her groceries this morning. When talking about her needs her pressing concern was for company, “I can’t imagine living alone”. When asked what her hopes were, she said to remarry. Attempted capacity assessment, patient was unable to retain information and so today would not be deemed to have capacity. She did state that her house was too large and mentioned that she should have taken the residential care option she considered a few years ago. Currently her level of care would be EMI social but a dual registered home may be advisable as she has retained social skills.

These notes show how documentation can be quite different, but easily deficient as regards definite evidence that the criteria of the MCA have been satisfied. In the end, Mrs. Gardiner went home and was felt to have capacity, albeit this was judged as borderline.

Generally, healthcare professionals across disciplines acknowledged that it was important to document decisions clearly, particularly in complex cases. Tensions exist, however, between the benefits of detailed, systematic, structured recording and the resource implications attached to increased documentation. In addition, the changing clinical picture may make it difficult for the formal recording of capacity to keep up with events. The recognition of the need for careful note-keeping, however, is frequently not met in practice.

### Patient narratives

Assessments about capacity and best interests are strongly influenced by the complex stories that build up around patients during their admissions. We observed value judgements being made about the reliability of the information provided by the patient with dementia. Particular events – a very elderly patient saying that her mother will look after her at home, a relative or friend describing risky behaviour, or a specific clinical finding, such as a low score on a cognitive test – might trigger doubts about capacity [23]. To such triggers will be added ‘collateral’ histories, from relatives, community-based practitioners and from previous inpatient admissions. These multiple and sometimes conflicting histories, which healthcare professionals have to interpret and make sense of, verify or falsify the patient’s version of events and influence subsequent judgements about capacity and best interests.

The extracts below illustrate contrasting narratives from Mrs. Carter and her daughter. The OT meanwhile uses a practical assessment to establish which account seems more plausible. The OT report was considered by the MDT to provide firmer evidence of capacity (despite in itself being a practical assessment of skills and not actually a capacity assessment).

Interview with Mrs. Carter

[….did you have anyone come in to help you when you lived at home?]“No”

[No, did nobody come into help you with…]

“Well it’s only small and I’ve only got the one bedroom. I’ve got a bedroom, bathroom, sitting room and kitchen, so I can manage all that.”

Interview with Mrs. Carter’s daughter

[So how often was someone going in to assist with her meals and medication?]

“They were going in and she was getting like three times a day for medication and like (Grandson 1) was going and (Grandson 2) was going nearly every night to put her meals out; but I mean, as I said, she wasn’t eating them, but she was getting thinner and thinner do you know”

Interview with OT 01BsD-1709

“But it became very clear, on her home visit, apart from the physical aspect, we do visit, ‘how do you do meals?’ Even if we know the answer we would ask. ‘I do all my own cooking, I cook from fresh, I do…’ And she doesn’t and as I've said before the family have supported her a lot. She struggled with making a hot drink and that was something that she had been doing sort of up to a couple of months before she came into hospital, and then it sort of deteriorated. She wasn’t managing. Couldn’t figure out how many she was making, how full she had to fill the kettle. She did say ‘What happens now? What are we doing?’”

Typically, the medical notes embody the ‘official’ patient narrative. Mrs. Salter’s daughter described at interview how, in a discharge planning meeting, a temporary OT and social worker, who had little prior knowledge of her mother, made their judgements based upon the story recorded in the medical records. The daughter felt this was unfair.

… she’d found out a little bit, but it was as if she’d read a book, picked out those bits of information about my mother and just was ready to deliver it.

… And even the second time, … even then it was still negative, you know; ‘Well I’m concerned about such-and-such, and such-and-such’; no interest in the whole patient; it’s just, I’ve got this information from the documentation and I’m going to read it out for you, …

Interview with family carer – Mrs Salter’s daughter

Understanding the patient’s narrative is obviously relevant to deciding on best interests. But the narrative also forms the background to decisions about capacity by, for instance, creating the impression of incompetence.

The theme of narrative raises further questions about the authority of different accounts. Our observation is that it is quite frequently true that a particular account (rightly or wrongly) dominates the narrative relevant to the judgement that has to be made. This, in turn, will often reflect power relations and, in most cases, the older person with dementia is the least likely to be consulted about what may or may not be in their best interests. Once a doubt has been triggered, despite the principle enshrined in the MCA that capacity should be presumed, their accounts are prone to be disbelieved. Ward observations confirmed the tendency for there to be ‘malignant positioning’ of people with dementia; that is, the labels of dementia or cognitive impairment can themselves make it more likely that decisions will undermine the standing of the person as such [24].

For instance, Mrs. Friar’s home was originally described as: 'like a circus … like a madhouse'. She was described by a junior doctor as being ‘not all there’; and both Mr. and Mrs. Friar were described as ‘weird’. The household was described as on the point of ‘social breakdown’. Following a home visit, however, the predominant narrative changed and the descriptions of both Mr. and Mrs. Friar were altered. She was then felt to have capacity and she went home. This case also raises issues about the timing of assessments.

### Time and timing

Many questioned the appropriateness of assessments of residence capacity in an acute hospital environment. This theme related both to getting the timing of assessments right in terms of the patient’s trajectory and to the opportunity to build a holistic view of the patient over time.

Ward-based observations show that informal and formal assessments of capacity can occur at any point and can vary over time. Professionals and families expressed concerns about fluctuations in capacity, especially in borderline cases, which make it imperative that decisions are not made at the wrong time.

The field note below suggests how a busy, noisy and sometimes chaotic ward, with a lack of privacy, can be the wrong environment for a careful capacity assessment. The right time also requires the right environment.

…the lost old age psychiatry referral was causing kind of a lot of noise in the room. The ward sister and the consultant were talking quite loudly about this missing referral, looking through the patient’s notes while the registrar was sat down on her haunches next to the patient, talking to her about her memory, asking about concerns about going home, if her family had any concerns. The patient said she was aware that her family had concerns but she didn’t have any problems at night. The registrar explained to her that her son had some concerns before the patient was admitted and it was really hard after that to hear what the patient was saying …, and it just struck me … it must have been quite difficult for the registrar and the patient. I feel the registrar is trying to assess the patient’s capacity and it must have been quite distracting for the patient. I found it very distracting.

Field notes 02–090309: Ward round

Healthcare professionals suggested the use of rehabilitation wards or step-down units for complex assessments and decisions of this magnitude.

INT: I think time, you need plenty of time to sit down and give them time. I mean elderly medicine consultations can’t be hurried. I think that resource is important … But is an acute hospital bed the right environment to do all that? … I’m not sure, because you expose them to hospital acquired infections. And so I think once their medical issues are resolved, they should be in a place where, like a rehab place or a community place where they are given time, family’s given time and their capacity, best interests are explored in detail and then made arrangements to comply with both.

Interview with consultant 01BsE-1610

## Discussion

### Principal findings

Our principal finding is that assessments of residence capacity, consequent judgements about best interests and subsequent discharge decisions are complex from every perspective: difficult for healthcare professionals, but crucial for people with dementia (because determinative of their human rights) and for their families. This complexity occurs in connection with decisions that are required daily and which might be regarded as routine. It can be understood in terms of five themes. First, borderline capacity requires careful assessment, which can be resource-intensive, but where all involved are required to grapple with the problems which emerge when evaluative decisions are required, especially where values are diverse and conflicting.^c^ Of course, those cases where capacity was not considered to be borderline, where the decisions about capacity just were more straightforward, could still be complex when it came to discharge (as in the case of Mr. Ryder). Again, this can reflect the importance of diverse and conflicting values. Secondly, we have shown the tendency for professional and family carers to focus on outcome (e.g. issues around risk and safety) rather than on the functional nature of capacity. This is understandable, because there is a natural concern to be beneficent and avoid harm to older people with dementia; but it flies in the face of the law’s protection of the individual’s right to self-determination, as seen in the recent legal case *CC v KK and STCC* [2012] EWHC 2136 (COP). Thus, whilst the healthcare and social care teams may be very concerned about safety at home, the patient simply has to demonstrate – as part of the functional assessment – that they are aware of the risks but have weighed them up and are willing to take them. The tendency amongst many professionals is to conflate the assessment of capacity and judgements about best interests, which is achieved in part by taking an outcomes approach to the determination of residence capacity. Furthermore, under such circumstances, the teams should in any case offer support at home to make matters as safe as possible, whilst respecting the patient’s wishes. One upshot of the outcomes approach is that it becomes highly likely that a finding of incapacity as regards residence will mean that the person does not go home, as evidenced by our research. Even where cases are straightforward, where capacity is not borderline, there need be neither the presumption that capacity means the person should go home, nor the presumption that incapacity means they should go into care. Thirdly, we demonstrated the difficulties around documentation: not only was this routinely poor, but how best to make it both suitably thorough and practicable is a challenge. Fourthly, we have helped to show the importance of (formal and informal) narrative. There are questions about the authenticity of any particular account and why some stories seem more salient and some story-tellers more authoritative. Finally, the issue of time is crucial: both the timing of and time allowed for assessments and decisions which are, after all, of immense importance to the person and to his or her family. Complex decisions about residence capacity and best interests seem not to be most suitably made in busy acute medical wards. Of course, practicalities may mean that there is no option but to assess residence capacity in such settings; but, if so, those involved need to be aware of the dangers of doing so because of the deeply evaluative nature of such assessments [1]. A quick assessment, without the necessary time for the sort of iterative process that capacity assessments might require, could compromise basic human rights. One helpful possible solution would be if patients had previously appointed a proxy to make decisions for them (under the MCA this would involve a Lasting Power of Attorney (LPA)). But a capacity assessment would still be required, for if the person had the capacity the attorney would be irrelevant. In any case, in the UK at least, there remain a number of barriers to any form of advance care planning, including the use of LPAs [25].

### Strengths and weaknesses

The strengths of this research stem from its ethnographic methods, which allowed us to capture the breadth and depth of daily processes on medical wards. The detail of the observations and data captured from multiple perspectives allowed us insight into the personal experiences of both those involved in making, and those on the receiving end of, such decisions. Our follow-up interviews with patients and carers at three months has helped to emphasize the importance of these decisions. Decisions about residence capacity and best interests are taken for granted as part of daily health and social care practice, but they have an enormous impact on patients and families. One measure of our objectivity is shown by the tendency of our results to be critical of practice despite our previous, and continuing, close relationships with, and high regard for, the professionals who were observed. Even in jurisdictions which do not use the ‘best interests’ standard, the quality of the decisions on the person’s behalf still need to be of a high order to protect his or her rights. To our knowledge there are no similar studies looking at the actual practice of decisions about residence capacity and best interests. Yet, as *CC v KK* shows, practice in this area is vital for the person’s human rights.

Although we think our research methods were robust in terms of both the number of cases and the variety of data collected, there may yet be limitations in terms of generalizability. It could be that similar research in other contexts (e.g. in different geographical regions or specialities) or at other times might yield different results. For instance, reflecting the area in which the research was carried out, the ethnic mix of the sample studied was very limited. Further research involving greater ethnic diversity might have a significant impact on our conclusions. Although we set out to study best interests as well as the determination of residence capacity, our focus has mainly been on the latter in this paper. Our intention would be to consider best interests in more detail in a future publication. Nonetheless, we have commented in this paper on the paucity of detailed recording of best interests decisions, the importance of a narrative account in determining best interests and the manner in which capacity assessments seem to predict decisions about outcome, because capacity and best interests are conflated by the use of an outcomes approach to capacity assessments. In addition, the MCA is now more deeply embedded in practice, so its implementation may well be different. However, recent evidence to the House of Lords Select Committee on the MCA has suggested that the principles of the Act are still far from deeply ingrained in practice [8]. Our subjective impression is that if things have changed they have not changed significantly and we are unaware of any research which might suggest otherwise (see also [26]).

### Other studies

Other studies have explored the practical application of the MCA in old age psychiatry services in relation to how capacity assessments are carried out and documented for community patients [27]. There is a strong association between the likelihood of discharge to care and a finding of incapacity [28]. Like us, Mujic et al. found that disagreement with the clinical team on the part of the patient was likely to lead to an assessment of incapacity [28]; they suggested that some medical teams were applying a *status* approach to the assessment of capacity, i.e. regarding the presence of dementia as itself indicative of incapacity. Researchers have considered the attitudes of clinicians and referral patterns to Independent Mental Capacity Advocate (IMCA) services [29], which has helped to highlight the importance of placement and discharge decisions. Much of this research has included older people generally and not solely people with dementia. Brown et al. looked retrospectively at a large number of capacity assessments in psychiatric admissions and showed that, whilst the number of assessments has increased since the MCA came into force, they were inconsistent and did not use the MCA’s criteria adequately [26]. But the study was not confined to older people and neither specified exactly how many had dementia nor how many assessments were for residence capacity. There is a broad body of literature around capacity, best interests and hospital discharge for older patients, sometimes with dementia [30-32]. But there is relatively little literature looking specifically at residence capacity and best interests [33,34]. Relevant issues do arise in connection with discharge planning [35,36]; and both residence capacity and best interests are discussed more theoretically in connection with ethics [37-39]. It is striking, however, given its ubiquity in clinical practice, how infrequently residence capacity is specifically discussed in the literature.

### Implications for practice

Each of our five themes carries implications for practice. The complexity of borderline decisions means that we need more support and training for practitioners, as well as support for the patients and families involved. In particular, this will require attention to issues around communication emphasized by values-based practice [40]. More specific training is still required around the MCA and the importance of functional as opposed to outcome assessments of capacity. As a specific example of this, we saw very little evidence of “all practicable steps” being taken to help people with dementia to participate in decisions being made about them, as required in Section 1(3) of the MCA. In one case a referral was sent to the speech and language department; this was not specifically for decision-making, but was for communication difficulties generally. Our results suggest that, on the whole, lower MMSE scores, which might be regarded as standing proxy for severity of illness, predict a lack of capacity. But for individuals this is by no means certain (cf. Mr. Saddler and Mr. Collier in Table 2). The MMSE neither determines residence capacity, nor any other specific capacity [41]. We also feel that crucial to the assessment of residence capacity is the need to be clear about the information to be imparted to the person concerned and we have made suggestions about its content [6]. Advocacy to support people with dementia facing decisions about place of residence might be required, not just for those who are unsupported and currently receive the support of an IMCA. It may be that families require more specific support too, because of their crucial role in the patient’s narrative. Finding documentation that is fit for purpose will remain important, but the deeper issue is that of embedding the appropriate assessments into routine clinical practice, for instance by using appropriately constructed care pathways. It may be that local guidelines could establish a proforma that should be used and help to set minimum standards for assessment and recording. Ideas around narrative underpin clinical practice broadly and raise questions about the authority of any particular account, but a specific issue is that the views of the person with dementia should be centre-stage. One aspect of this is that the person with dementia should as far as possible be involved in decisions about him or herself, especially in connection with decisions about best interests. People with dementia were sometimes included in MDT meetings, but not always. Sometimes they wanted to be but were not; on other occasions they did not wish to be included in the meetings but were. The wishes of people with dementia are not always assessed appropriately. Similarly, about three patients from the rehabilitation ward and one from an acute ward were taken to see the care homes that were being considered for them. But it was not clear how this fed into the decision-making about their final placement. Mr. Collier, for instance, was taken to see one care home on the other side of the city to where he had lived, to which he was then sent. It was Mr. Collier who talked, at follow-up, about being tricked. Finally, the issue of time suggests to us the need for properly resourced step-down or rehabilitation units. A recent meta-analysis of rehabilitation for older adults showed improvements in functional outcomes, with lower mortality and fewer admissions to institutional care [42].

One more general issue, with specific relevance to the use of the MCA, is to what extent the language of the MCA (or relevant capacity legislation elsewhere) is being used to effect the outcome that is desired. This is not just the point about functional as opposed to outcome approaches to assessment. The MCA was intended both to enable individuals who lack capacity and to protect those who have to make decisions for them. It may be that in some areas, whilst protecting professionals, it is not being put into effect in a way that enables individuals to retain as much control as possible over decisions that face them. In a focus group for professionals a higher trainee in geriatric medicine said the following:

… but I don’t feel that it happens in real life really, I don’t. I think if the MDT and the patient’s relatives decide that they should, that their level of requirement is that they might need care, I don’t feel that we do assess their capacity. If they just kind of, if patients are placid as you call it, if there’s no big objection, if they’re not saying loudly “I want to go home”, then I don’t feel that on a routine basis we assess their capacity to agree with us, we only assess their capacity if they don’t.

**Professional Focus Group *280409:829–834***

This is an admission that capacity is assessed for the purposes of the professionals, to sanction their decisions, not to enhance the autonomy of the patients. The doctor in the focus group quickly added: ‘I’m not saying that’s the right thing…’. It is more that there are sociological or environmental factors weighing on professionals, which encourage them to use assessments to bring about outcomes that are good for the system as a whole rather than being good for the individuals concerned.

### Implications for future research

The issue of documentation is crucial, because this could help to guide decision-making through the complexity of the clinical, ethical and legal processes. Documentation would need to be part of a care pathway, which itself would need to be embedded in the right sort of approaches and attitudes in practice. Effecting change in clinical practice involves multiple, complex, dynamic, professional and social adjustments affecting different layers of an organization [43,44]. Hence, further research on how change might be achieved with respect to the practice of assessing residence capacity and best interests seems important and necessary. More theoretically, the issue of narrative raises the possibility of conceptual links between our themes, person-centred care and the law around capacity, best interests and deprivation of liberty. Specific developments in the law, either in terms of case law, or in terms of changes to (e.g.) the MCA’s Code of Practice [45], may well be worthy of further applied research.

## Conclusions

This in-depth ethnographic exploration of twenty-nine patient cases highlights the complexity of judgements around residence capacity and best interests. We have identified specific themes that suggest areas for practice improvement. This will involve professional training and the possibility of greater legal safeguards for people with dementia facing discharge from hospital, as well as resource implications. The moral and legal imperative is such, however, that it seems difficult not to accept that changes in practice are required.

## Endnotes

^a^It is a fundamental point that capacity is always assessed in connection with a specific decision. This research was specifically about residence capacity, i.e. about the person’s ability to make decisions about where they should live. We have not always stipulated, when using the word ‘capacity’, that we have been considering ‘residence capacity’ but this will normally be the case, or we hope that the context will make it plain that some other sense of “capacity” is at issue.

^b^We are aware that not every jurisdiction operates according to the ‘best interests’ principle and that this can, in any case, be interpreted differently. Nevertheless, if the person is found to lack the requisite capacity or to be ‘incompetent’, a decision still has to be made about what should be done on his or her behalf. At this point the threat to the person’s safety or to their rights becomes very real if the wrong decision is made. And it is with this that the present paper is concerned.

^c^The topic of borderline capacity and its relevance need further discussion. We are preparing a paper to discuss this theme in more detail.

## Abbreviations

COP: Court of Protection; EMI: Elderly mentally infirm; IMCA: Independent Mental Capacity Advocate; LPA: Lasting power of attorney; MCA: Mental capacity act; MDT: Multidisciplinary team; MMSE: Mini-Mental State Examination; NHS: National Health Service; OT: Occupational therapist; SpR: Specialist Registrar (higher training grade in the UK); UK: United Kingdom.

## Competing interests

The authors declare that they have no competing interests.

## Authors’ contributions

MP reviewed the literature, undertook the ethnographic observation, analysed and interpreted the data, and drafted the manuscript. JB designed the study, formulated the research question, analysed and interpreted the data, helped in the collection of data, and revised the manuscript. CE helped to design the study, formulated the research question, reviewed the literature, contributed to data analysis and revised the manuscript. HG helped with data collection, reviewed relevant literature and revised the manuscript. SJL and LR helped to design the study, commented on data analysis, and revised the draft. JCH designed the study, formulated the research question, reviewed the literature, helped to analyse and interpret the data, helped in the collection of data, and drafted the manuscript. JCH is guarantor. All authors read and approved the final manuscript.

## Authors’ information

MP is a post-graduate social science researcher (1); JB is professor of social gerontology and health services research (1); CE is a senior law lecturer and qualified solicitor (2); HG is a consultant in old age psychiatry and leads the Northern Regional Gender Dysphoria Service (3); SJL is a consultant physician in care of the elderly (4); LR is professor of primary care and ageing (1); JCH is a consultant in psychiatry of old age and honorary professor of philosophy of ageing (5, 6).

## Pre-publication history

The pre-publication history for this paper can be accessed here:

http://www.biomedcentral.com/1471-2318/14/56/prepub

## References

[B1] Greener H Poole M Emmett C Bond J Louw SJ Hughes JC Value judgements and conceptual tensions: decision-making in relation to hospital discharge for people with dementia Clin Ethics 2012 14 166 174 doi:10.1258/ce.2012.012028 10.1258/ce.2012.012028

[B2] Hughes JC Alzheimer’s and Other Dementias: The Facts 2011 Oxford: Oxford University Press

[B3] Department of Health Prime Minister’s Challenge on Dementia: Delivering Major Improvements in Dementia Care and Research by 2015 2012 London: Department of Health Available at: https://www.gov.uk/government/uploads/system/uploads/attachment_data/file/215101/dh_133176.pdf [last accessed 28th September 2013]

[B4] Sampson EL Blanchard MR Jones L Tookman A King M Dementia in the acute hospital: prospective cohort study of prevalence and mortality Br J Psychiatry 2009 14 61 66 doi:10.1192/bjp.bp.108.055335 10.1192/bjp.bp.108.055335 19567898

[B5] Alzheimer’s Society Counting the Cost: Caring for People with Dementia on Hospital Wards 2009 London: Alzheimer’s Society Available at: http://www.alzheimers.org.uk/site/scripts/documents_info.php?documentID=1199 [last accessed 28th September 2013]

[B6] Emmett C Poole M Bond J Hughes JC Homeward bound or bound for a home? Assessing the capacity of dementia patients to make decisions about hospital discharge: comparing practice with legal standards Int J Law Psychiat 2013 14 73 82 10.1016/j.ijlp.2012.11.009 23187119

[B7] Department of Health ‘Nothing Ventured, Nothing Gained’: Risk Guidance for People with Dementia 2010 London: Department of Health Available at: https://www.gov.uk/government/uploads/system/uploads/attachment_data/file/215960/dh_121493.pdf [last accessed 28th September 2013]

[B8] House of Lords Select Committee on the Mental Capacity Act 2005 – Report. Mental Capacity Act 2005: post-legislative scrutiny Available at: http://www.publications.parliament.uk/pa/ld201314/ldselect/ldmentalcap/139/13902.htm [Last accessed 25th April, 2014]

[B9] Hammersley M Atkinson P Ethnography: Principles in Practice 1995 2 London and New York: Routledge

[B10] Gergen KJ An Invitation to Social Construction 2009 2 London: Sage

[B11] Glaser B The constant comparative method of qualitative analysis Soc Probl 1965 14 436 445 10.2307/798843

[B12] Department of Health Guidance on nominating a consultee for research involving adults who lack capacity to consent 2008 London: Department of Health

[B13] Silverman D Interpreting Qualitative Data 2011 4 London: Sage

[B14] Walsh D Seale C Doing ethnography Researching Society and Culture 2012 2 London: Sage 245 262

[B15] Charmaz K Constructing Grounded Theory: A Practical Guide Through Qualitative Analysis 2006 London: Sage

[B16] NVivo Qualitative Data Analysis Software 2010 QSR International Pty Limited Version 9

[B17] Glaser BG Strauss AL The Discovery of Grounded Theory: Strategies for Qualitative Research 1967 Chicago: Aldine

[B18] Folstein MF Folstein SE McHugh PR ‘Mini-mental state’. A practical method for grading the cognitive state of patients for the clinician J Psychiatr Res 1975 14 189 198 10.1016/0022-3956(75)90026-6 1202204

[B19] Freedman B Competence, marginal and otherwise: concepts and ethics Int J Law Psychiat 1981 14 53 72 10.1016/0160-2527(81)90020-0 7327819

[B20] Fulford KWM Bill Radden J Facts/Values. Ten principles of values-based medicine The Philosophy of Psychiatry: A Companion 2004 Oxford: Oxford University Press 205 234

[B21] Emmett C Poole M Bond J Hughes JC Residence capacity: complexity and confusion Elder Law J 2013 14 159 166

[B22] Welsh SF Keeling A Jacob R, Gunn M, Holland A The deprivation of liberty safeguards Mental Capacity Legislation: Principles and Practice 2013 London: RCPsych Publications 78 95

[B23] Twining C Woods B, Clare L Capacity and consent: empowering and protecting vulnerable older people Handbook of the Clinical Psychology of Ageing 2008 Chichester: John Wiley & Sons 429 436

[B24] Sabat SR The Experience of Alzheimer’s Disease: Life Through a Tangled Veil 2001 Oxford: Blackwell

[B25] Robinson L Dickinson C Bamford C Clark A Hughes J Exley C A qualitative study: professionals’ experiences of advance care planning in dementia and palliative care, ‘*a good idea in theory but…*’ Palliat Med 2013 14 401 408 doi:10.1177/0269216312465651 10.1177/0269216312465651 23175508

[B26] Brown PF Tulloch AD Mackenzie C Owen GS Szmukler G Hotopf M Assessments of mental capacity in psychiatric inpatients: a retrospective cohort study BMC Psychiatry 2013 14 115 doi:10.1186/1471-244X-13-115 10.1186/1471-244X-13-115 23586975 PMC3643852

[B27] Shah A Banner N Heginbotham C Fulford B The application of the Mental Capacity Act 2005 among psychiatry patients: a pilot study Int Psychogeriatr 2009 14 922 930 doi:10.1017/S1041610209990391 10.1017/S1041610209990391 19552833

[B28] Mujic F von Heising M Stewart RJ Prince MJ Mental capacity assessments among general hospital inpatients referred to a specialist liaison psychiatry service for older people Int Psychogeriatr 2009 14 729 37 doi:10.1017/S104161020900917X 10.1017/S104161020900917X 19426580

[B29] Luke L Redley M Clare I Holland A Hospital clinicians’ attitudes towards a statutory advocacy service for patients lacking mental capacity: implications for implementation J Health Serv Res Policy 2008 14 73 78 doi:10.1258/jhsrp.2007.007084 10.1258/jhsrp.2007.007084 18416911

[B30] Chadwick R Russell J Hospital discharge of frail elderly people: social and ethical considerations in the discharge decision-making process Ageing Soc 1989 14 277 295 10.1017/S0144686X00013775 11659753

[B31] Kapp MB Decisional capacity in theory and practice: legal process versus “bumbling through” Aging Ment Health 2002 14 413 417 10.1080/1360786021000007054 12425775

[B32] Darzins P Can this patient go home? Assessment of decision-making capacity Aust Occup Ther J 2010 14 65 67 doi:10.1111/j.1440-1630.2010.00854.x 10.1111/j.1440-1630.2010.00854.x 20854566

[B33] Brindle N Holmes J Capacity and coercion: dilemmas in discharge of older people with dementia from general hospital settings Age Ageing 2005 14 16 20 10.1093/ageing/afh228 15496463

[B34] Stewart R Bartlett P Harwood R Mental capacity assessments and discharge decisions Age Ageing 2005 14 549 550 10.1093/ageing/afi185 16267176

[B35] Cooney LM Jr Kennedy GJ Hawkins KA Hurme SB Who can stay at home? Assessing the capacity to choose to live in the community Arch Intern Med 2004 14 357 60 10.1001/archinte.164.4.357 14980985

[B36] Carrese JA Refusal of care: patients’ well-being and physicians’ ethical obligations: “but Doctor, I want to go home” JAMA 2006 14 691 695 10.1001/jama.296.6.691 16896112

[B37] Strang DG Molloy DW Harrison C Capacity to choose place of residence: autonomy vs beneficence? J Palliat Care 1998 14 25 29 9575710

[B38] O’Keefe ST Autonomy vs welfare? Anatomy of a risky discharge Ir Med J 2001 14 234 246 11758623

[B39] Hughes JC Poole M Louw SJ Nudging the older person into care: an end to the dilemma? Am J Bioeth 2013 14 34 36 doi:10.1080/15265161.2013.781715 23641848 10.1080/15265161.2013.781715

[B40] Fulford KWM Peile E Carroll H Essential Values-Based Practice: Clinical Stories Linking Science with People 2012 Cambridge: Cambridge University Press

[B41] Sabat SR Capacity for decision-making in Alzheimer's disease: selfhood, positioning and semiotic people Aust N Z J Psychiatry 2005 14 1030 1035 doi:10.1080/j.1440-1614.2005.01722.x 10.1080/j.1440-1614.2005.01722.x 16343306

[B42] Bachman S Finger C Huss A Egger M Stuck AE Clough-Gorr KM Inpatient rehabilitation specifically designed for geriatric patients: A systematic review and meta-analysis of randomized controlled trials BMJ 2010 14 1718 doi:10.1136/bmj.c1718 10.1136/bmj.c1718 PMC2857746 20406866

[B43] Wood M Ferlie E Fitzgerald L Achieving clinical behaviour change: a case of becoming indeterminate Soc Sci Med 1998 14 1729 38 10.1016/S0277-9536(98)00250-0 9877343

[B44] Dopson S Fitzgerald L Knowledge to Action: Evidence-Based Health Care in Context 2005 Oxford: Oxford University Press

[B45] Department of Constitutional Affairs Mental Capacity Act 2005: Code of Practice 2007 London: Stationery Office Also available at: http://www.legislation.gov.uk/ukpga/2005/9/pdfs/ukpgacop_20050009_en.pdf (Last accessed 28th September 2013)

